# Evaluation of Hepatic Resection in Liver Metastasis of Gastric Cancer

**DOI:** 10.1007/s13193-018-0827-6

**Published:** 2018-11-22

**Authors:** Yukiko Nonaka, Kazuhiro Hiramatsu, Takehito Kato, Yoshihisa Shibata, Motoi Yoshihara, Taro Aoba, Tadahiro Kamiya

**Affiliations:** Department of General Surgery, Toyohashi Municipal Hospital, 50 Aza Hachiken Nishi, Aotake–Cho, Toyohashi, Aichi 441-8570 Japan

**Keywords:** Gastric cancer, Liver surgery, Liver metastasis, Management of metastasis

## Abstract

Gastric cancer is the second most common malignancy globally and the third most common cause of cancer-related deaths in Japan. In gastric cancer, benefit of surgical resection of liver metastasis, which was shown in colorectal cancer, is not well established. The present study aimed to examine the feasibility of hepatic resection for liver metastasis of gastric cancer. In this retrospective study, we reviewed the medical records of 10 patients with liver-only metastases of gastric cancer who underwent hepatectomy among 2043 patients with gastric cancer who underwent gastric resection between January and December 2016 at a single institution in Japan. Median 1-, 3-, and 5-year overall survival (OS) rates were 78.0%, 33.3%, and 22.2%, respectively, among 10 patients who underwent hepatic resection. There was a significant difference in OS rates between tumors measuring ≥ 5 cm and < 5 cm (hazard ratio [HR] 6.524, 95% confidence interval [CI] 1.145–37.171, *p* = 0.035). The longest survival was 205 months for one patient who was alive at the time of the analysis. Hepatic resection of liver metastasis in gastric cancer was associated with long-term survival in some patients. Additionally, primary tumor size was associated with long-term survival.

## Introduction

Gastric cancer is the second most common malignancy globally and the third most common cause of cancer-related deaths in Japan. Despite considerable advances in overall gastric cancer treatment, approaches for the treatment of gastric cancer metastasizing only to the liver have been controversial. Some studies suggest that hepatectomy is effective against gastric cancer with only liver metastasis, with a 5-year overall survival (OS) ranging from 20 to 40% [1–11], whereas results of other studies investigating the benefit of hepatic resection for liver metastasis of gastric cancer were unclear [12–14]. The present study aimed to evaluate the outcomes of surgical treatment for liver metastasis of gastric cancer.

## Materials and Methods

**Fig. 1:**
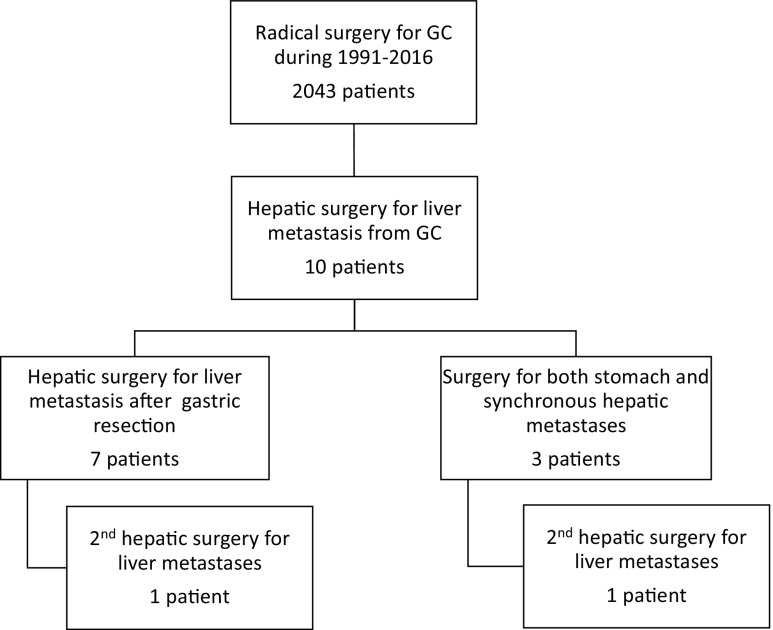
Flow chart

In this retrospective study, we reviewed the medical records of 10 patients with liver-only metastases of gastric cancer who underwent hepatic resection among 2043 patients with gastric cancer who underwent gastric resection between January and to December 2016 at Toyohashi Municipal Hospital. The study flowchart is presented in Fig. 1.

Inclusion criteria for this study were as follows: (1) histologically confirmed gastric cancer; (2) presence of synchronous or metachronous liver metastasis; and (3) surgical therapy performed between January 1, 1991, and December 31, 2017. Patients with double cancers were excluded. The study was conducted in accordance with the Declaration of Helsinki of 1975, revised in 2000. Clinical and pathological data included sex, age, and information regarding primary gastric cancer and liver metastases. The 14th edition of the Japanese Classification of Gastric Carcinoma was utilized for this study. Data regarding the last follow-up and vital status were collected for all the patients.

### Statistical Analysis

Univariate analysis was performed using Cox proportional hazards regression to identify the risk factors that were associated with OS and recurrence-free survival (RFS). All data were analyzed using the Statistical Package for Social Science software version 21.0 (SPSS, Chicago, IL, USA). For all analyses, *p* < 0.05 was considered to be statistically significant.

## Results

### Patient Characteristics

Patient characteristics are presented in Tables 1 and 2. In total, 10 patients, including nine males and one female, underwent hepatectomy for liver metastasis of gastric cancer. Synchronous and metachronous metastases were noted in four (40.0%) and six (60.0%) patients, respectively, whereas single and multiple liver metastases were found in seven (70.0%) and three (30.0%) patients, respectively. Tumor size was > 3 cm in four (40.0%) patients, and two patients (20.0%) had new liver metastases after hepatic resection and underwent second hepatic resection.

**Table 1:** Characteristics

Variable	Value
Age	
Median (range)	68 (47–75) years
Sex	
Male	9 (90.0%)
Female	1 (10.0%)
Tumor size	
< 5 cm	6 (60.0%)
≧ 5 cm	4 (40.0%)
Histological type	
Intestinal	6 (60.0%)
Diffuse	4 (40.0%)
Lymphatic invasion	
ly 0	2 (20.0%)
ly 1/2/3	8 (80.0%)
Tumor invasion	
T0/T1	1 (10.0%)
T2 SS	2 (20.0%)
T3 SE T4 SI	7 (70.0%)
Lymph node metastasis	
N0 + N1	7 (70.0%)
N2 + N3	3 (30.0%)
Metachronous/Synchronous	
Metachronous 6 (60.0%)	
Synchronous 4 (40.0%)	
Number of metastasis	
1	7 (70.0%)
2	3 (30.0%)
Number of hepatic surgery	
Once	8 (80.0%)
Twice 2	(20.0%)

**Table 2:** Clinical and pathological data

Patient	Age	Sex	Depth of tumor	Type of gastric cancer	Tumor size of gastric cancer [cm]	Number of lymph node metastasis	Level of lymphotic	Level of venous invasion	Operation type	TMN	Stage	
1	70	M	SS	Poor	2.5 × 2.5	2/22	1	2	Total gastrectomy	T3N1M1	4	
2	55	M	SM	Mode	2.5 × 2.5	0/17	0	0	Total with splenectomy	T1bN0M0	1A	
3	69	M	MP	Mode	2.5 × 2.2	2/18	2	1	Total with splenectomy	T2N0M0	1B	
4	67	M	SS	Papi	5.7 × 5.7	0/22	2	2	Distal gastrectomy	T3N0M0	2A	
5	65	M	MP	Well	2.3 × 2.3	2/22	1	1	Distal gastrectomy	T2N1M0	2A	
6	47	M	SE	Poor	9.0 × 7.5	13/16	3	2	Distal gastrectomy	T4aN3aM0	3C	
7	53	M	SS	Mode	4.0 × 4.3	6/23	2	2	Distal gastrectomy	T3N2M0	3A	
8	75	M	SS	Poor	3.0 × 3.5	0/2	0	2	Completion gastrectomy	T3N0M1	4	
9	75	F	SE	Well	10.0 × 6.5	7/23	3	3	Total gastrectomy with splenectomy and pancreatomy	T4aN3aM1	4	
10	74	M	SE	Poor	5.0 × 5.0	2/14	2	2	Distal gastrectomy	T4aN1M1	4	
Patient	Type of liver metastasis			Number of hepatic metastasis	Tumor size of hepatic meta [cm]	Interval between operations [month]	Operation type		Survival time after hepatectomy [month]	Survival status	NAC	Adjuvant
1	Syn			1	1.5	12	Partial		11	Alive	SP	TS-1
2	Meta			2	2.5, 0.6	33	Partial		33	Death	–	–
3	Meta			1	2.5	36	Partial		33	Death	–	TS-1
4	Meta			2	3, 4	7	Partial		26	Death	–	–
5	Meta			1	4.5	59	Segmentectomy		39	Death	–	–
6	Meta			1	7	13	Partial		5	Death	–	FU/LV
7	Meta			1	4	7	Segmentectomy		205	Alive	–	UFT
8	Syn			1	0.8	0	Enucleation		62	Death	–	–
9	Syn			2	1.6, 0.8	0	Partial		10	Death	–	UFT
10	Syn			1	1	0	Partial		33	Death	–	UFT

### Patient Outcomes

Median age at the time of hepatic resection was 68 (range, 47–75) years. Patient outcomes are presented in Table 3. Briefly, 1-, 3-, and 5-year OS rates after surgery were 78.0%, 33.3%, and 22.2%, respectively, with a median OS of 2.583 years. Additionally, 1-, 3-, and 5-year RFS rates were 44.4%, 22.2%, and 22.2%, respectively, with a median RFS of 0.792 years.

**Table 3:** Univariate analysis

Univariate analysis of hazard ratio estimated by Cox regression (OS/RFS)							
	Number	HR	95% CI	*p*	HR	95% CI	*p*
Sex							
Female	1	1			1		
Male	9	0.118	0.007–1.886	0.131	0.407	0.042–3.931	0.437
Age years							
< 65	3	1			1		
≧ 65	7	1.493	0.296–7.529	0.628	1.345	0.264–6.854	0.721
Tumor size of primary cancer							
< 5 cm	6	1			1		
≧ 5 cm	4	6.524	1.145–37.171	0.035	3.216	0.706–14.649	0.131
Histological type							
Intestinal	6	1			1		
Diffuse	4	1.084	0.256–4.592	0.913	0.794	0.188–3.356	0.754
Lymphatic invasion							
ly0/ly1	4	1			1		
ly2/ly3	6	1.636	0.384–6.962	0.505	1.692	0.401–7.149	0.474
Venous invasion							
V0/V1	3	1			1		
V2/V3	7	0.939	0.206–4.288	0.935	0.555	0.123–3.507	0.444
Tumor invasion							
T0/T1/T2	3	1			1		
T3/T4	7	4.288	0.849–21.661	0.078	2.248	0.500–10.097	0.291
Lymph node metastasis							
N0	4	1			1		
N1/N2/N3	6	0.88	0.217–3.566	0.858	0.559	0.136–2.292	0.419
Metachronous/synchronous							
Metachronous	6				1		
Synchronous	4	0.98	0.231–4.160	0.978	1.345	0.264–6.854	0.721
Number of metastasis							
1	8	1			1		
2	2	3.369	0.658–17.237	0.145	3.609	0.592–22.011	0.164
Maximum size of the metastatic tumor							
< 3 cm	6	1			1		
≧ 3 cm	4	0.834	0.912–3.626	0.809	0.831	0.201–3.635	0.831
Hepatic surgery							
Once	8	1			1		
Twice	2	2.002	0.362–11.073	0.426	1.453	0.280–7.549	0.657

There was a significant difference in OS between patients whose primary gastric cancer measured ≥ 5 cm and those whose primary gastric cancer measured < 5 cm (hazard ratio [HR] 6.524, 95% confidence interval [CI] 1.145–37.171, *p* = 0.035). There was a trend towards a difference in OS rates between patients whose cancer depth was serosal exposure (SE) or serosal invasion (SI) and those whose cancer depth was shallower than SE (HR 4.288, 95% CI 0.849–21.661, *p* = 0.078).

## Discussion

Despite improved postoperative outcomes in recent years [15], survival rates of patients with liver metastasis of gastric cancer have not increased as much as those observed with hepatic resection for metastatic lesions of colon cancer [12–14]. Liver metastasis is an important point of consideration in treatment regimens for patients with gastric cancer [16, 17]. Compared with colon cancer, fewer patients with gastric cancer are candidates for hepatic resection as they often harbor multiple liver metastases as well as coexisting metastases in other locations.

Several recent studies on liver resection in gastric cancer reported that some patients achieved long-term survival of 2–6 years, with 1- and 5-year OS rates of 60–77% and 10–42%, respectively, and a median survival time ranging from 8.8 to 34 months; the findings of the current study are in agreement with these previous reports [1–11].

Among many studies investigating prognostic factors for liver resection in gastric cancer, several reported that single liver metastases and those measuring < 5 cm were associated with good prognosis [2–5, 9]. However, in the present study, the number of liver metastases and tumor were not associated with OS. Patients with single liver metastatic lesions died within a year, and there was only one patient whose tumor diameter was 5 cm. The patients in the present study were stratified according to a tumor diameter of 3 cm. Further analysis using a tumor diameter cutoff value of 4 cm did not reveal significant differences in OS rates between the groups (data not shown).

Our results suggest that primary gastric cancer invading deeper than submucosa may be a poor prognostic factor. Serosal invasion is a proposed mechanism of peritoneal seeding [15], and some studies suggest that serosal invasion may be a poor prognostic factor for liver surgery [8, 10]. In the present study, there were two patients with primary gastric cancers that were not deeper than submucosa who survived for 33 and 39 months, respectively. Of the remaining eight patients, six (75%) died within 3 years of hepatic resection. The depth of primary gastric cancer should be considered as a prognostic factor in liver metastasis of primary gastric cancer.

Although several prognostic factors were reported to be associated with liver metastasis of primary gastric cancer, the size of primary gastric cancer was not previously reported as a significant predictor of favorable outcomes, which should be evaluated in future studies.

In the current study, two of the 10 patients underwent hepatic resection twice, and both survived for > 2 years after the first hepatic resection, longer than the reported survival time. Surgery should be considered with consideration of the patient’s clinical condition for recurrent liver metastasis following the first hepatic resection in patients with gastric cancer.

One major limitation of the present study is its retrospective design that involved a single institution; therefore, the number of patients was small, and only univariate analysis was performed. However, our cohort is similar to most studies on liver metastasis of gastric cancer which included a series of 10–20 patients at most, with a very limited number of larger cohorts available at this time. Despite the limited number of studies on hepatic resection of liver metastasis in gastric cancer, this approach was reported to be ineffective in certain patients. Therefore, future, large-scale studies are necessary to identify those patients who should undergo surgery.

## Conclusion

Some of the patients undergoing hepatic resection for liver metastasis of gastric cancer achieved long-term survival. Primary tumor size was associated with long-term survival.
